# Prevalence of bacteriospermia and its association with semen parameters among men attending a fertility clinic in Kumasi, Ghana

**DOI:** 10.3389/frph.2026.1853404

**Published:** 2026-07-10

**Authors:** Evans Anokye Kumi, Victor Boachie Owusu, Ebenezer Kojo Addae, Peter Nyarko Coffie, Eric Darko, Rex Kwadwo Mawuli Djokoto, Kweku Bedu-Addo, John Asiedu Larbi

**Affiliations:** 1 Department of Theoretical and Applied Biology, Kwame Nkrumah University of Science and Technology, Kumasi, Ghana; 2 Department of Nursing, Valley View University, Kumasi, Ghana; 3 Department of Physiology, School of Medicine and Dentistry, Kwame Nkrumah University of Science and Technology, Kumasi, Ghana; 4 Oak Specialist Hospital, Kumasi, Ghana; 5 Department of Physiology, Accra College of Medicine, Accra, Ghana; 6 Department of Clinical Microbiology, Kwame Nkrumah University of Science and Technology, Kumasi, Ghana; 7 Department of Obstetrics and Gynaecology, Komfo Anokye Teaching Hospital, Kwame Nkrumah University of Science and Technology, Kumasi, Ghana

**Keywords:** bacteriospermia, Ghana, infertility, prevalence, semen quality

## Abstract

**Background:**

Male infertility is a growing reproductive health concern in Ghana, contributing significantly to the burden of infertility among couples. Bacteriospermia, the presence of bacteria in semen, has been implicated in male infertility through its potential adverse effects on sperm quality; however, its association with semen parameters remains inconsistent across studies. This study aimed to determine the prevalence of bacteriospermia and examine its association with semen parameters among males attending a fertility clinic in Kumasi, Ghana.

**Materials and methods:**

Semen samples from 226 males attending a fertility hospital in Kumasi were collected and evaluated according to WHO guidelines. Additionally, samples were cultured for bacteria using standard bacterial culture techniques.

**Results:**

The prevalence of bacteriospermia was 46.5%, with *Escherichia coli* (41.9%), *Klebsiella* spp. (25.7%), *Staphylococcus*spp. (24.8%), *Streptococcus* spp. (2.9%), and *Bacillus* spp. (1.9%) identified. Abnormal semen parameters were observed in 47.6% of participants, predominantly teratozoospermia. No significant association was found between bacteriospermia and semen parameters (*p* > 0.05), though semen volume approached significance as a predictor of bacteriospermia (*p* = 0.057), with hypospermia associated with significantly lower odds of bacteriospermia (aOR = 0.558; *p* = 0.047) and hyperspermia showing no significant association (aOR = 0.961; *p* = 0.160).

**Conclusions:**

A high prevalence of bacteriospermia (46.5%) and abnormal semen parameters (47.6%, predominantly teratozoospermia) were observed among men seeking fertility evaluation, with *E. coli* as the most frequently isolated organism. No significant association was found between bacteriospermia and semen parameters, though hypospermia was associated with significantly lower odds of bacteriospermia. Routine screening and further research incorporating molecular techniques and larger cohorts are recommended.

## 1 Introduction

Infertility is increasingly recognized as a significant public health issue, affecting about 10% of couples globally (1), with male factors accounting for 20%–30% of cases (2). Male infertility, specifically, is the failure of a male to impregnate a fertile female after a year of unprotected intercourse (3). The prevalence of bacteriospermia in infertile men ranges from 15% to 35.3% (4, 5). In Africa, the prevalence rates of specific conditions contributing to male infertility include oligospermia (31%), asthenozoospermia (19.39%), and varicocele (19.2%) (6). In Ghana, the prevalence of male infertility is an emerging concern, with estimates ranging from 12% to 17% (7, 8). A study by Blay et al. found male infertility to be more prevalent than female infertility, with rates of 15.8% and 11.8%, respectively (9).

Bacteriospermia is the presence of bacteria in semen. Clinically defined as bacterial count exceeding 1,000 colony-forming units (CFU)/mL in ejaculate, bacteriospermia affects semen quality in both humans and animals (10). Common bacteria found in cases of bacteriospermia include *Escherichia coli (E.Coli)*, *Enterococcus faecalis, Streptococcus agalactiae, and Staphylococcus aureus* (4, 5). The presence of these microorganisms in sperm potentially contributes to complications such as decreased sperm quality, motility, and overall infertility in males (11).

Bacteriospermia is clinically important because it may impair male fertility through multiple mechanisms, including disruption of spermatogenesis, reduced sperm function, oxidative stress-induced DNA damage, induction of anti-sperm antibodies, and inflammation-related obstruction of the male reproductive tract (12). Pathogenic bacteria in ejaculates can negatively impact sperm count, morphology, and motility, which are key clinical indicators of male reproductive health. The disruption of spermatogenesis and semen function, as well as the blockage of the urogenital tract, may result from direct bacterial interaction and the involvement of immune-competent cells (11). However, the specific impact of each pathogen on seminal parameters remains unclear. Some studies indicate that bacterial infections can harm the male urogenital system, while others argue that such infections do not significantly alter sperm characteristics (13). Emerging seminal microbiome studies suggest that the relationship between semen-associated bacteria and male fertility is complex, with some bacterial species potentially exerting neutral or context-dependent effects on semen quality rather than being strictly pathogenic (14, 15).

Despite the rising prevalence of male infertility in Ghana, few studies have investigated the specific bacterial pathogens present in semen and their potential effects on semen quality. Understanding the microbiological profile of semen is crucial for improving diagnostic and therapeutic approaches in male infertility. Culture-based methods were employed in this study as they remain the gold standard for detecting clinically significant infections and are readily applicable in routine clinical practice, particularly in resource-limited settings. The study aimed to identify the bacterial species in semen and assess their influence on semen parameters among men attending a fertility clinic in Kumasi, contributing valuable data to guide diagnosis and management of male infertility in similar contexts.

## 2 Methodology

### 2.1 Study area

This study was conducted at Oak Specialist Hospital, a HeFRA-accredited fertility clinic in the Kumasi Metropolis, Ghana, offering comprehensive reproductive health evaluation and management in compliance with Ghana Medical and Dental Council guidelines.

### 2.2 Study design

**Figure 1:**
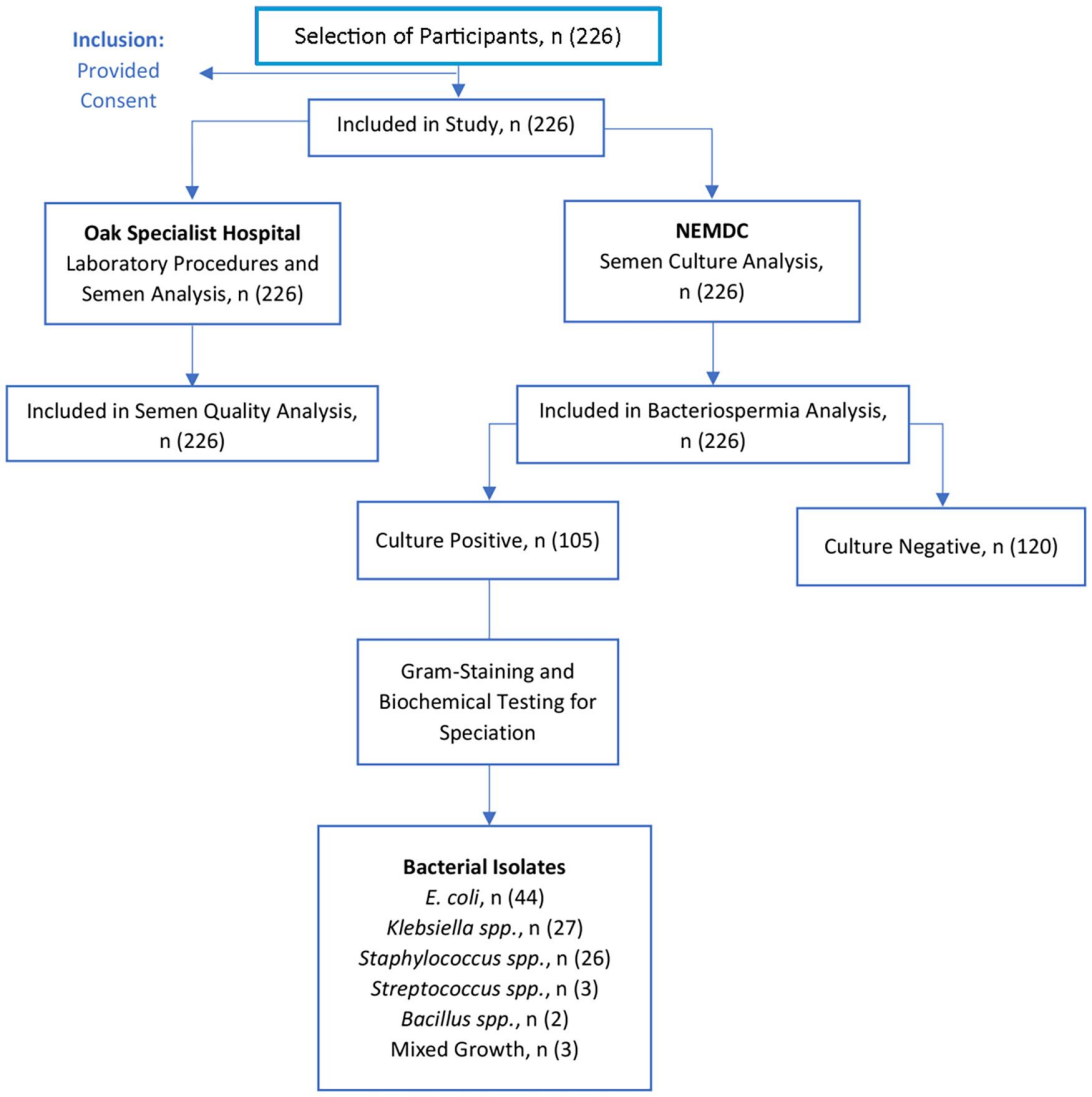
Flow chart of participant recruitment and data analysis.

This hospital-based cross-sectional study was conducted from February 2024 to July 2024 at Oak Specialist Hospital, a leading fertility centre in Kumasi, Ghana. The study included male patients attending a fertility clinic for fertility evaluation who provided informed consent. Participants were not classified as infertile or fertile, as definitive clinical infertility diagnosis was not established for all individuals. Exclusion criteria included individuals who declined participation, patients with known systemic conditions (e.g., diabetes, hypertension) or localised disorders (eg., varicocele) known to affect semen parameters. Also, patients with recent use of medications that could alter semen quality (e.g., antibiotics, hormonal therapies) and those with azoospermia were excluded. Semen samples were analysed at Oak Specialist Hospital for standard semen parameters following WHO guidelines. For microbiological assessment, samples were transported under optimal conditions to the New Era Medical and Diagnostic Centre (NEMDC) in Kumasi within 30–45 min of collection for bacterial culture using standard microbiological techniques (Figure 1).

#### 2.2.1 Sample size

The sample size was calculated using Cochran's formula for an unlimited population.

Unlimited population (N): $\frac{Z^{2}X\hat{\;p}\;(1-\hat{\;p})}{\varepsilon^{2}}$

Where z is the Z score (1.96), *ε* is the margin of error (5%), N is the population size). p^ is the population proportion [assumed as 15.8% according to (9)], *n* is the number from the unlimited population. Substituting the variables into the formula yields a minimum sample size of 205.

### 2.3 Data collection

Structured questionnaires were administered to collect socio-demographic data from participants. Demographic data collected included age, occupation and risk of occupation, and marital status, while laboratory data included semen quality parameters (Sperm Morphology, concentration, motility, and Semen volume) and semen abnormalities. We categorized the various occupations based on their relative risk of exposure to factors that might impact semen parameters such as heat, toxins, sedentary behaviour and stress (16, 17): High-Risk occupations included miners, electricians, sonographers, mechanics and spare parts dealers; Moderate-Risk included mechanical engineers, contractors, drivers and farmers; Low-Risk included bankers, police officers, businessmen, journalists, teachers and lecturers, health workers etc.

### 2.4 Laboratory procedures and analysis

#### 2.4.1 Semen collection and analysis

Semen samples were collected in sterile containers through masturbation, following the recommended abstinence period (2–7 days) as per WHO guidelines (18). Participants were instructed to wash their hands and genital area thoroughly with clean water before sample collection. Various semen parameters, including appearance, volume, pH, liquefaction, count, motility, morphology, and concentration, were analysed. All analyses were conducted in accordance with WHO standard protocols, ensuring consistency and accuracy in evaluating semen quality (19). Semen parameters were classified according to World Health Organization (19, 20) reference criteria. Oligozoospermia was defined as sperm concentration <15 million/mL. Asthenozoospermia was defined as progressive motility <32% or total motility <40%. Teratozoospermia (abnormal morphology) was defined as <4% normal sperm forms. These thresholds were used to categorize semen abnormalities for statistical analysis. The parameters were assessed using standardized laboratory techniques, as detailed below.

##### 2.4.1.1 Appearance

After liquefaction, the appearance of semen samples was evaluated by inspecting their color at room temperature. Any alterations in visual appearance, such as changes in color, clarity, or the presence of mucous streaks, were noted.

##### 2.4.1.2 Volume

The volume of each semen sample was measured using a graduated measuring cylinder, with the volume recorded in milliliters.

##### 2.4.1.3 ph

Semen pH was determined using a narrow-range pH paper (pH 6.4–8.0). A drop of the sample was evenly spread onto the pH paper (Sperm Processer, India), and the color change was compared to a calibrated strip after 30 s.

##### 2.4.1.4 Concentration

Sperm concentration was assessed using a Makler counting chamber (Sefi-Medical Instruments, Italy). The semen sample was diluted, loaded onto the chamber, and sperm cells were counted under a microscope, with the concentration calculated using the chamber's specific formula.

##### 2.4.1.5 Motility

Total motility was assessed by placing a 10 μl drop of well-mixed, liquefied semen onto a counting chamber and examining it within 3–5 min under a light microscope at ×200 magnification. Sperm motility was classified according to WHO criteria as progressive motility, non-progressive motility, and immotile sperm.

##### 2.4.1.6 Sperm morphology

A semen smear was prepared for morphological assessment. Clean glass slides were washed in 70% ethanol and air-dried in a sterile rack. A 5 µl aliquot of well-mixed semen was applied to each slide. Smears were fixed in 95% ethanol for 5–10 min and air-dried. They were then treated with sodium bicarbonate–formalin solution to remove mucus and rinsed with running water. The smears were stained sequentially using diluted carbol fuchsin and crystal violet for 2 min, followed by washing with water. Lugol's iodine was applied for 1 min as a mordant and rinsed with distilled water. Counterstaining was performed using dilute Loeffler's methylene blue–safranin (0.1%) for 2 min, after which slides were washed, drained, and air-dried. Stained slides were examined under a light microscope using ×40 and ×100 (oil immersion) objective lenses. Sperm head, midpiece, and tail abnormalities were evaluated, and 100 spermatozoa were assessed per slide.

### 2.5 Microbiological analysis

Semen samples were cultured on Blood agar and MacConkey agar prepared according to the manufacturer's instructions. All samples were inoculated within 1 h of collection and incubated aerobically at 37 °C for 24–48 h. Bacterial growth was quantified by colony-forming units per milliliter (CFU/mL), and organism identification was performed using standard microbiological procedures, including Gram staining and biochemical tests. Gram-positive cocci (*Staphylococcus* spp. and *Streptococcus* spp.) and Gram-positive bacilli (*Bacillus* spp.) were identified using Gram staining and standard biochemical tests. *Staphylococcus* spp. were further confirmed using catalase and coagulase tests. Gram-negative organisms (*Escherichia coli* and *Klebsiella* spp.) were identified using indole, citrate utilization, oxidase, and urease tests where appropriate. Mixed growth was recorded when more than one organism was isolated from a single sample and analyzed separately. In this study, bacteriospermia was defined as a bacterial concentration of ≥1 × 10³ CFU/mL, consistent with thresholds commonly used in andrological and microbiological studies to distinguish clinically relevant bacterial presence from contamination. Samples with counts below this threshold were considered negative (20). Mixed cultures were interpreted with caution to distinguish probable contamination from clinically significant polymicrobial growth based on culture characteristics and laboratory criteria.

### 2.6 Statistical analysis

Data was entered using Microsoft Excel 2019 (Microsoft Inc., USA) and analysed using GraphPad Prism 8.0.2 (263) (GraphPad Software, Inc, San Diego, CA, USA). Descriptive statistics were used to summarize the prevalence of bacterial presence and the distribution of semen parameters within the study population. Fisher's exact test and binary logistic regression were employed to evaluate the association between bacterial presence and semen parameters, with odds ratio calculated using the Baptista-Pike method. Statistical significance was set at a *p*-value of <0.05 with a 95% confidence interval.

### 2.7 Ethical considerations and approval

The study was conducted in accordance with the principles outlined in the Declaration of Helsinki. Ethical approval was obtained from the Committee on Human Research, Publications, and Ethics (CHRPE) of Kwame Nkrumah University of Science and Technology, with reference number CHRPE/AP/107/24. Informed, written consent was obtained from all participants prior to their inclusion in the study.

## 3 Results

The study selected a total of 226 study participants with median age of 40 years (IQR: 34–46 years) and age ranging from 26 to 64 years. Majority of participants were found in the cohort 26–35 years (68, 30.1%), followed by the 36–40 years (51, 22.6%) and 41–45 years (47, 20.8%). Additionally, 24.8% (56) of participants were engaged in moderate risk occupations such as engineers, contractors, drivers, farmers, while 10.2% (23) were engaged in high-risk occupations (miners, electricians, mechanics, sonographers, spare parts dealers). Most of the participants were married (221, 97.8%) as illustrated in Table 1.

**Table 1:** Prevalence of semen abnormalities among study participants.

Demographics	Frequency (*N* = 226)	Percent (%)
Age Group (Years)		
26–35	68	30.1
36–40	51	22.6
41–45	47	20.8
46–50	31	13.7
51–64	29	12.8
Occupation risk on semen quality		
Low risk	147	65.0
Moderate risk	56	24.8
High risk	23	10.2
Marital Status		
Married	221	97.8
Unmarried	5	2.2

**Figure 2:**
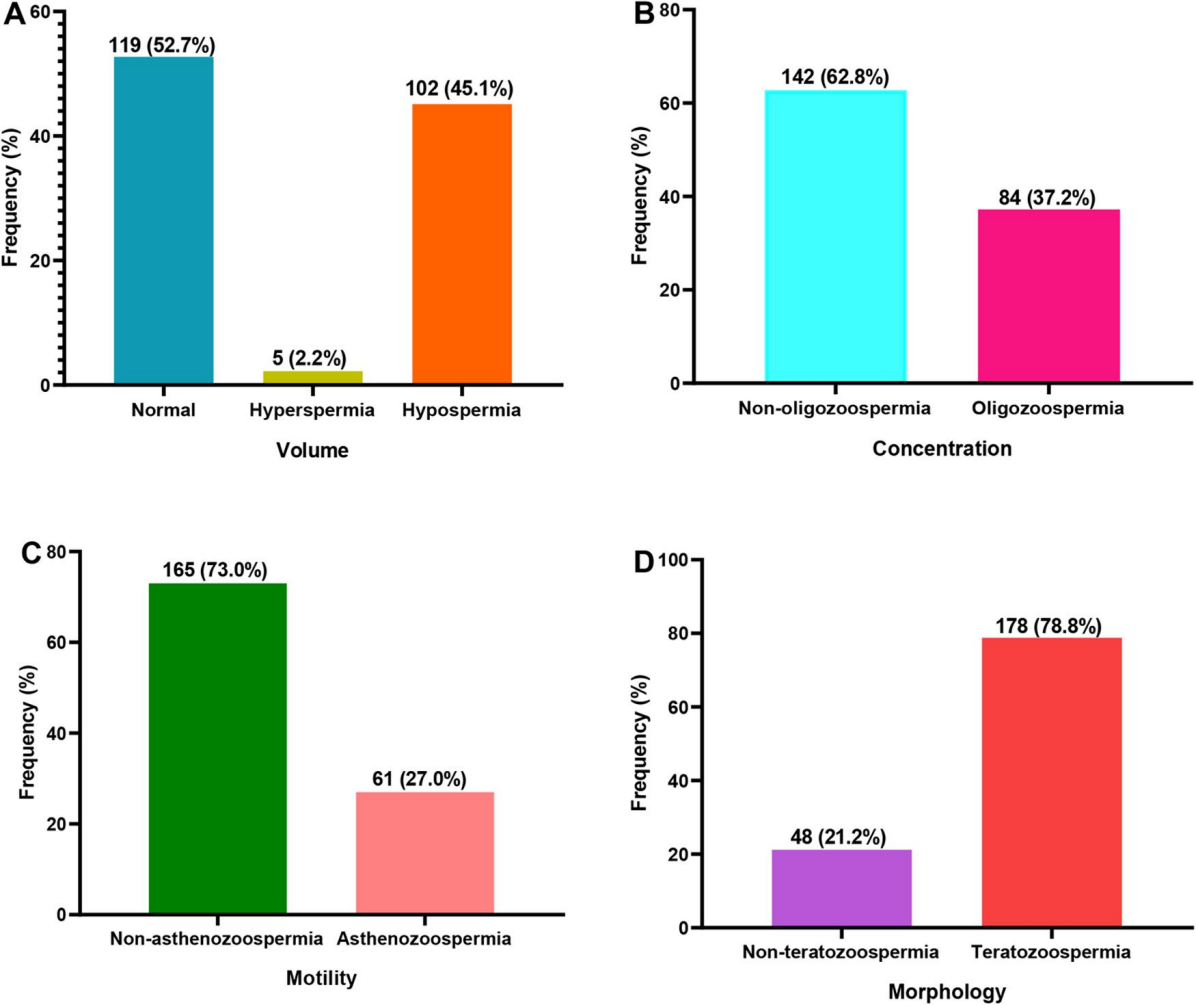
Prevalence of semen abnormalities among study participants. **(A)** Semen volume categories showing normal semen volume, hyperspermia, and hypospermia. **(B)** Sperm concentration categories showing non-oligozoospermia and oligozoospermia. **(C)** Sperm motility categories showing non-asthenozoospermia and asthenozoospermia. **(D)** Sperm morphology categories showing non-teratozoospermia and teratozoospermia. Bars are labeled with the corresponding frequencies and percentages.

Routine semen analysis revealed that 119 (52.7%) of men had normal semen volume, 102 (45.1%) were hypospermic, and 5 (2.2%) were hyperspermic. Most of the participants, 142 (62.8%) had normal semen concentration without oligozoospermia. Majority of the participants, 165 (73.0%) exhibited normal motility with no asthenozoospermia, whereas 61 (27.0%) showed evidence of asthenozoospermia. Teratozoospermia was observed in 178 participants (78.8%), while 48 (21.2%) had normal sperm morphology (Figure 2).

### 3.1 Prevalence of bacteriospermia and bacteria species among study participants

**Figure 3:**
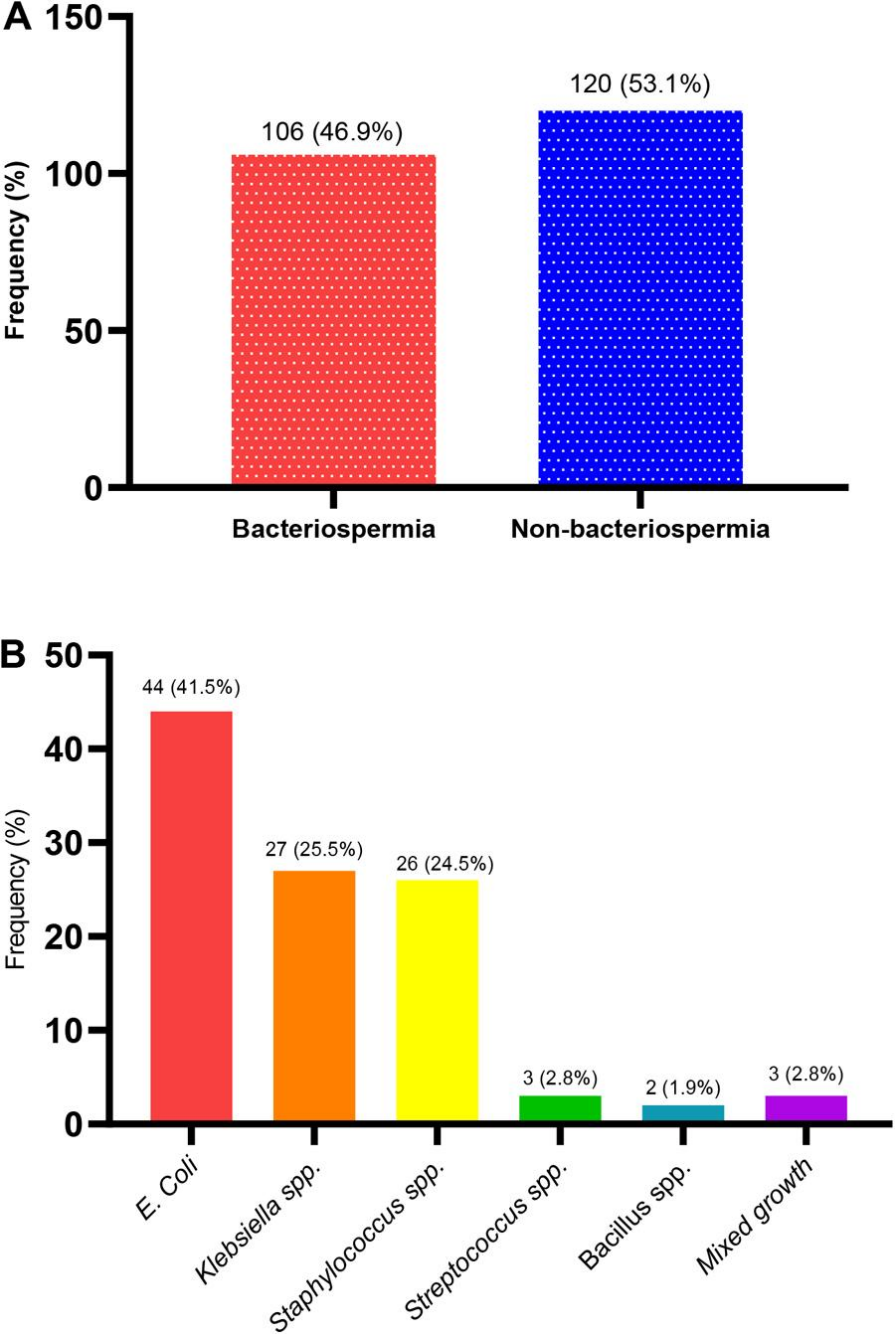
Prevalence of bacteriospermia and bacterial species among study participants. **(A)** Distribution of participants with bacteriospermia and non-bacteriospermia. **(B)** Distribution of bacterial species isolated from semen samples, including Escherichia coli, Klebsiella spp., Staphylococcus spp., Streptococcus spp., Bacillus spp., and mixed bacterial growth. Bars are labeled with the corresponding frequencies and percentages.

Of the 226 samples, 105 (46.5%) showed bacterial growth (1 × 10³ to 3 × 10³ CFU/mL) with significant colony count, while 121 (53.5%) had counts below 1 × 10³ CFU/mL, classified as negative cultures by WHO criteria. Five bacterial species were isolated: *E. coli* 44 (41.9%), *Klebsiella spp*. 27 (25.7%), *Staphylococcus spp.* 26 (24.8%), *Streptococcus spp*. 3 (2.9%), *Bacillus spp.* 2 (1.9%), and mixed growth 3 (2.9%) (Figure 3).

Fisher's Exact Test revealed no statistically significant association between bacteriospermia and sperm morphology (*p* = 0.420) (Table 2). In the adjusted binary logistic regression model, bacteriospermia was not an independent predictor of teratozoospermia (Wald *χ*² = 0.207, df = 1, *p* = 0.649; OR = 1.181), explaining only 1.1% of the variance (Nagelkerke R² = 0.011). Although men with teratozoospermia had 18.1% greater odds of bacteriospermia compared with those with normal morphology, this association was statistically non-significant (Table 3).

**Table 2:** Effect of bacteriospermia and bacterial isolates on sperm morphology.

Variable	Teratozoospermia	
	cOR (95% CI)	*p*-value
Bacteria presence		
Negative	0.75 (0.39–1.43)	0.42
Positive		
Isolates		
*E. coli*	0.89 (0.38–2.22)	0.82
*Klebsiella spp.*	0.89 (0.35–2.27)	0.80
*Staphylococcus spp.*	1.42 (0.48–3.81)	0.61
*Streptococcus spp.*	-Inf (0.27-inf)	> 0.99
*Bacillus spp.*	0.30 (0.02–6.00)	0.42
Mixed growth	0.62 (0.07–9.26)	0.56

-Inf indicates infinity. Fischer's exact test was used to compute significance.

**Table 3:** Binary logistic regression analysis of sperm morphology and bacteriospermia.

Variable	aOR (95% CI)	*p*-value
Constant	0.922 (0.854−1.086)	0.570
Non-Teratozoospermia vs. Teratozoospermia	1.181 (0.987–1.275)	0.649

Non-teratozoospermia (reference), teratozoospermia; aOR, adjusted odds ratio; CI, confidence interval. Model adjusted for waist circumference, hip circumference, occupation, smoking status, mobile phone proximity to groin, dietary sugar intake, and alcohol consumption.

Fisher's Exact Test revealed no significant association between bacteriospermia and sperm motility (*p* = 0.23) (Table 4). In the adjusted binary logistic regression model, sperm motility was not an independent predictor of bacteriospermia (Wald *χ*² = 2.131; OR = 0.960, df = 1, *p* = 0.144), explaining 1.1%–1.5% of the variance (Nagelkerke R² = 0.011–0.015). Although men with asthenozoospermia had approximately 4% lower odds of bacteriospermia compared with those with normal motility, this association was statistically non-significant (Table 5).

**Table 4:** Effect of bacteriospermia and bacterial isolates on sperm motility.

Variable	Asthenoteratozoospermia	
	cOR (95% CI)	*p*-value
Bacteria presence		
Negative	0.67 (0.38–1.21)	0.23
Positive		
Isolates		
*E. coli*	1.53 (0.62–3.76)	0.48
*Klebsiella spp.*	1.63 (0.59–4.34)	0.43
*Staphylococcus spp.*	0.54 (0.18–1.73)	0.42
*Streptococcus spp.*	n/c	> 0.99
*Bacillus spp.*	n/c	> 0.99
Mixed growth	n/c	> 0.99

n/c indicates not calculable. Fischer's exact test was used to compute significance.

**Table 5:** Binary logistic regression analysis of sperm motility and bacteriospermia.

Variable	aOR (95% CI)	*p*-value
Constant	0.931 (0.749–1.008)	0.618
Non-Asthenozoospermia vs. Asthenozoospermia	0.960 (0.938–1.01)	0.144

Non-asthenozoospermia (reference), asthenozoospermia; aOR, adjusted odds ratio; CI, confidence interval. Model adjusted for waist circumference, hip circumference, occupation, smoking status, mobile phone proximity to groin, dietary sugar intake, and alcohol consumption.

No significant association was observed between sperm concentration and bacteriospermia using Fisher's Exact Test (*p* = 0.210; Table 6). In the adjusted binary logistic regression model, sperm concentration was not an independent predictor of bacteriospermia (aOR = 1.365; Wald *χ*² = 1.102, df = 1, *p* = 0.295), explaining only 0.6%–0.8% of the variance (Nagelkerke R² = 0.006–0.008), indicating a negligible predictive contribution (Table 7).

**Table 6:** Effect of bacteriospermia and bacterial isolates on sperm concentration.

Variable	Oligozoospermia	
	cOR (95% CI)	*p*-value
Bacteria presence		
Negative	0.69 (0.39–1.18)	0.21
Positive		
Isolates		
*E. coli*	0.39 (0.15–1.14)	0.14
*Klebsiella spp.*	1.04 (0.45–2.73)	> 0.99
*Staphylococcus spp.*	0.54 (0.19–1.34)	0.33
*Streptococcus spp.*	1.03 (0.07–9.10)	> 0.99
*Bacillus spp.*	2.09 (0.11–40.22)	0.55
Mixed growth	4.31 (0.48–63.23)	0.25

Fischer's exact test was used to compute significance.

**Table 7:** Binary logistic regression analysis of sperm concentration and bacteriospermia.

Variable	aOR (95% CI)	*p*-value
Constant	0.756 (0.652–1.26)	0.240
Non-oligospermia vs. Oligospermia	1.365 (1.268–1.643)	0.295

Non-oligospermia (reference), oligospermia; aOR, adjusted odds ratio; CI, confidence interval. Model adjusted for waist circumference, hip circumference, occupation, smoking status, mobile phone proximity to groin, dietary sugar intake, and alcohol consumption.

No significant association was observed between semen volume and bacteriospermia using Fisher's Exact Test (*p* = 0.060; Table 8). In the adjusted binary logistic regression model, semen volume approached but did not reach statistical significance as a predictor of bacteriospermia (Wald *χ*² = 5.731, df = 2, *p* = 0.057), explaining 2.9%–3.8% of the variance (Nagelkerke R² = 0.029–0.038). Compared with men with normal semen volume, those with hypospermia had significantly lower odds of bacteriospermia (aOR = 0.558; *p* = 0.047), whereas hyperspermia was not significantly associated with bacteriospermia (aOR = 0.961; *p* = 0.160) (Table 9).

**Table 8:** Effect of bacteriospermia and bacterial isolates on semen volume.

Variable	Hyperspermia		Hypospermia	
	cOR (95% CI)	*p*-value	cOR (95% CI)	*p*-value
Bacteria				
Negative	0.00 (0.00–0.68)	0.06	0.58 (0.34–1.00)	0.06
Positive				
Isolates				
*E. coli*	n/c	> 0.99	1.59 (0.70–3.62)	0.31
*Klebsiella spp.*	n/c	> 0.99	1.10 (0.45–2.76)	0.82
*Staphylococcus spp.*	n/c	> 0.99	0.78 (0.33–2.01)	0.65
*Streptococcus spp.*	n/c	> 0.99	0.78 (0.05–6.85)	> 0.99
*Bacillus spp.*	n/c	> 0.99	n/c	> 0.99
Mixed growth	n/c	> 0.99	n/c	> 0.99

n/c indicates not calculable; Fischer's exact test was used to compute significance.

**Table 9:** Binary logistic regression analysis of semen volume and bacteriospermia.

Variable	aOR (95% CI)	*p*-value
Constant	1.229 (0.979–1.330)	0.288
Hypospermia vs. Normal	0.558 (0.493–0.601)	0.047
Hyperspermia vs. Normal	0.961 (0.864–1.010)	0.160

Normal (reference); hypospermia, hyperspermia; aOR, adjusted odds ratio; CI, confidence interval. Model adjusted for waist circumference, hip circumference, occupation, smoking status, mobile phone proximity to groin, dietary sugar intake, and alcohol consumption.

## 4 Discussion

Semen analysis continues to be the most effective and essential diagnostic tool, identifying infertility issues in 9 out of 10 men with genuine male infertility problems (21). The key measurable attributes of semen—total sperm count and the volume of fluid produced by accessory glands—alongside the characteristics of sperm (such as vitality, motility, and morphology) and the composition of seminal fluid, are crucial for proper sperm function (19). This study assessed the quality of semen and the presence of bacteria in males attending fertility hospitals in Kumasi.

Among the various semen parameters examined, teratozoospermia emerged as the most prevalent abnormality, affecting 78.8% of the participants. This finding is slightly lower than, 81.17%, in a previous study (12), but significantly higher than the 18.5% and 23.33%, observed by Owolabi et al. and Agyepong and Bedu-Addo respectively (18, 22), indicating variability in the presence of teratozoospermia across different populations and settings. Oligospermia was observed in 37.2% of the semen samples, closely aligning with the 39.33% prevalence reported by an earlier study in Ghana (18). This similarity suggests that oligospermia may have a consistent prevalence in certain populations, possibly influenced by common environmental or genetic factors. Asthenozoospermia had the lowest prevalence in this study, affecting 27.0% of participants. This finding is consistent with the similar prevalence reported by (18). The lower occurrence of asthenozoospermia relative to other abnormalities may reflect different underlying causes or risk factors, which could vary by region or population.

In this study, bacteriospermia was found in 46.5% of men, a prevalence rate consistent with findings by Osazuwa et al. and Cottell et al. (23, 24) who reported rates of 52.5% and 51% respectively. These similarities suggest that bacteriospermia is a common issue among men attending fertility clinics, possibly due to similar environmental or clinical factors across different populations. However, findings contradict that of Agyapong and Bedu-Addo (18), Vilvanathan et al. and Domes et al. (12, 25), who reported lower prevalence rates of bacteriospermia, 22.3%, 35.3% and 15% respectively. The discrepancies may be attributed to differences in study populations, geographic locations, and diagnostic techniques.

The present study found that bacteriospermia did not significantly impact semen parameters. This observation aligns with the findings of Domes et al. and Cottell et al. (24, 25), but contrasts with the results of Isaiah et al. (26), Golshani et al. (27) and Agyapong and Bedu-Addo (18). One reason for this discrepancy could be that not all bacterial species are equally pathogenic, meaning they may have minimal or no detectable effect on semen parameters. Additionally, the bacterial load, or concentration, might be too low in some cases to significantly impair semen quality. Aurich et al. (28) demonstrated that the effect of bacteria on cooled-stored stallion spermatozoa varied depending on the bacterial species and load, while Grande et al. (29) emphasized that the impact of seminal microbiota on male infertility is complex and influenced by both the pathogenicity and concentration of the bacteria. Furthermore, the male reproductive system has compensatory mechanisms that can maintain semen quality despite bacterial infections. Johnson et al. (30) noted that increased spermatogenesis or enhanced epididymal sperm maturation might counteract any negative effects of bacteria. Washburn et al. (31) further explained that the resilience of spermatogenesis can mitigate defects caused by bacterial infections. Another factor could be the subclinical or transient nature of some bacterial infections, which might resolve before causing significant damage to semen quality. While bacteria may be present, the duration and intensity of the infection may not be sufficient to significantly affect semen parameters. Zeyad et al. (32) concluded that bacteria in semen might not always lead to detectable changes in semen characteristics, especially if the infections are transient. Similarly, Cottell et al. (24) questioned the significance of seminal fluid microorganisms, suggesting that many could be contaminants rather than pathogens.

In the adjusted binary logistic regression model, semen volume was examined as a predictor of bacteriospermia after adjusting for relevant covariates. Overall, semen volume was not a statistically significant predictor of bacteriospermia, although the association approached significance, with the model explaining approximately 2.9%–3.8% of the variation in bacteriospermia status. Notably, a meta-analysis by Pergialiotis et al. found that total sperm volume was not significantly affected by the presence of bacteriospermia when all pathogens were analyzed together, which is consistent with the weak and borderline association observed in the present study. Compared with men with normal semen volume, those with hypospermia had lower odds of bacteriospermia. However, given the borderline overall model significance and the small proportion of variance explained, this association should be interpreted cautiously. This finding may partly reflect the aetiology of hypospermia itself. Low ejaculate volume is frequently caused by mechanical or structural conditions such as ejaculatory duct obstruction or retrograde ejaculation rather than genital tract infection (33), and men with hypospermia in this sample may have had predominantly non-infectious causes of reduced semen volume, which could partly explain the lower odds of bacteriospermia observed. In ejaculatory duct obstruction specifically, hypospermia arises because the seminal vesicles — which contribute approximately 70%–80% of normal ejaculate volume — are excluded from the ejaculate due to anatomical obstruction rather than bacterial activity (34).

Furthermore, reduced ejaculate volume may also reflect decreased contribution from accessory gland secretions, although the extent to which these influences bacterial colonization remains unclear and warrants further investigation. In contrast, hyperspermia was not significantly associated with bacteriospermia. Given the lack of statistical significance, no clear relationship between increased semen volume and bacteriospermia could be established in this cohort. Hyperspermia, characterized by an ejaculate volume exceeding 5.5 mL, has not been consistently associated with impaired sperm function (35), and the present findings suggest it similarly does not meaningfully alter the likelihood of bacterial presence. The biological mechanisms underlying this finding remain unclear, and further studies are needed to determine whether variations in accessory gland secretions influence bacterial colonization of semen. Taken together, these findings are consistent with the observation by Grande et al. (29) that the influence of seminal microflora on male fertility is shaped by biological context and bacterial characteristics, suggesting that semen volume alone is not a strong determinant of bacteriospermia status.

The bacterial isolates identified in this study—*E. coli*, *Staphylococcus spp.*, and *Klebsiella spp.*—were comparable to those found in the descriptive study conducted by (18), as well as (12), who as well identified *E. coli*, *Staphylococcus*, *Klebsiella*, and other bacteria. In our study, *E. coli* was the most prevalent bacterium (41.9%), followed by *Klebsiella* (25.7%), *Staphylococcus* (24.8%), and *Streptococcus* (2.9%). The high prevalence of *E. coli* and *Staphylococcus* is consistent with the findings of (18), who also recorded *E. coli* and *S. aureus* as the predominant bacterial isolates. *Enterococcus faecalis* to be the most dominant bacterial isolate, followed by *Staphylococcus*, with *Klebsiella* being among the less frequently isolated bacteria (12). This contrasts with our study, where *Klebsiella* was the second most predominant bacterial isolate. The discrepancies may be attributed to differences in study populations and geographic locations.

## 5 Conclusions

This study highlights a high burden of male fertility abnormalities among men attending fertility clinics, with 47.6% exhibiting abnormal semen parameters, predominantly teratozoospermia, and bacteriospermia identified in 46.5% of participants, with *Escherichia coli* as the most frequently isolated organism. No statistically significant association was observed between bacteriospermia and semen parameters. Semen volume approached but did not reach significance as a predictor of bacteriospermia, with hypospermia associated with significantly lower odds of bacteriospermia and hyperspermia showing no significant association, suggesting semen volume alone is an insufficient determinant of bacteriospermia status. These findings should be interpreted with caution given the cross-sectional design, limited statistical power, and unmeasured confounders including bacterial virulence factors, host immune responses, and oxidative stress. Future studies incorporating molecular microbiological techniques, inflammatory markers, and larger well-characterized cohorts are recommended.

### 5.1 Recommendations

The high prevalence of bacteriospermia supports continued microbiological evaluation in men undergoing fertility assessment. Future studies should:

1. Standardize and validate diagnostic thresholds, ideally using species-specific cutoffs or sensitivity analyses.
2. Integrate molecular techniques, such as 16S rRNA sequencing, alongside conventional culture to detect non-culturable organisms.
3. Use longitudinal designs with repeated semen sampling and larger, diverse populations to capture temporal variability and better clarify clinical relevance.
4. Incorporate leukocyte quantification and inflammatory biomarkers since leukocytes may contribute to oxidative stress and sperm dysfunction.
5. Future studies should include a well-defined fertile control group to enable comparative analysis of bacteriospermia prevalence and its association with semen parameters between fertile and infertile men.

### 5.2 Limitations

First, semen analysis was based on a single time-point measurement, which may not fully capture intra-individual variability in semen parameters. Semen quality is known to fluctuate due to physiological, environmental, and lifestyle factors; therefore, a single sample may not be fully representative of an individual's typical reproductive profile.

Second, bacteriospermia was defined using a uniform colony-forming unit (CFU) threshold (≥1 × 10³ CFU/mL). While this cutoff is consistent with commonly used definitions in the literature, there is no universally accepted standard, and the clinical significance of bacterial presence may vary across species. The use of a single threshold may therefore oversimplify species-specific pathogenic effects and could influence the interpretation of associations observed in this study.

Third, bacterial identification was performed using conventional culture-based microbiological methods. Although these techniques remain the clinical gold standard for detecting viable and clinically relevant organisms, they may underestimate the full spectrum of seminal microbiota by failing to detect fastidious or non-culturable bacteria. This limitation may be particularly relevant in the context of predominantly null findings, as undetected microbial populations could contribute to unobserved associations.

The study did not assess bacterial virulence factors such as adhesins, toxins, or biofilm-forming ability, which may influence the pathogenic potential of isolates and their effects on semen quality.

The exclusion of azoospermic men may introduce selection bias, as bacteriospermia prevalence and microbial profiles may differ in this subgroup and were not assessed in this study.

## Data Availability

The raw data supporting the conclusions of this article will be made available by the authors, without undue reservation.
